# Optimum electronic structures for high thermoelectric figure of merit within several isotropic elastic scattering models

**DOI:** 10.1038/s41598-017-10511-x

**Published:** 2017-08-30

**Authors:** Yuli Yan, Yu Rong Jin, Guangbiao Zhang, Jiong Yang, Yuanxu Wang, Wei Ren

**Affiliations:** 1 Institute for Computational Materials Science, School of Physics and Electronics, Henan University, Kaifeng, 75004 People’s Republic of China; 2 Department of Physics, International Center for Quantum and Molecular Structures, and Materials Genome Institute, Shanghai University, Shanghai, 200444 China

## Abstract

Electronic band structure is vital in determination the performance of thermoelectric materials. What is the optimum electronic structure for the largest figure of merit? To answer the question, we studied the relationship between the thermoelectric properties and the electronic band structure under the assumption of isotropic elastic scattering, within the context of Chasmar-Stratton theory. The results show that whether the anisotropic band structure and the effective mass of the carrier are beneficial to improving the thermoelectric properties. The scattering mechanism and the shape of the Fermi surface play a decisive role. Regardless of scattering mechanism type, a larger valley degeneracy is always beneficial to thermoelectric materials.

## Introduction

One possible way to improve the figure of merit (ZT) of a material is to reduce its lattice thermal conductivity by enhancing phonon scattering such as by the filler atoms, by dimensionality reduction or by introducing fine nano-inclusions into the bulk matrix^1–5^. However, a very comprehensive review done by Spitzer shows that 0.2 W/(m.K) is the practical lattice thermal conductivity lower limit for semiconductors^6^. Therefore, further improving ZT can only be achieved through improvement of the thermoelectric power-factor (S^2^ *σ*). The decoupling of S, *σ*, and *κ*_*e*_ in an effort to achieve a high ZT has been a longstanding challenge as they are strongly coupled with each other through the phonon and electron scattering and band structure. According to the Chasmar-Stratton theory, the optimal electronic performance of a thermoelectric semiconductor depends primarily on the weighted mobility, µ${({{\rm{m}}}^{\ast })}^{\frac{3}{2}}$, here m* is the density of states effective masses, *μ* is the mobility of charge carriers^7–9^. Typically, each degenerate carrier pocket makes a contribution to m* via m* = ${N}_{v}^{\frac{2}{3}}{{\rm{m}}}_{b}$ (m_*b*_ the average band mass) and m_*b*_ = ${({m}_{x}{m}_{y}{m}_{z})}^{\frac{1}{3}}$ for an anisotropic material in three principle directions having effective mass of *m*_*x*_, *m*_*y*_, and *m*_*z*_ (in unit of the free mass m_0_, m_0_ = 9.11 × 10^−31^ kg is the free electron rest mass). *N*_*v*_ is the degeneracy of the band extrema near the Fermi level^7^.

Several theories have been put forward as to optimize electrical properties and improve thermal-to-electricity conversion efficiencies of thermoelectric materials^10–13^. But these theories are mainly based on the acoustic phonon scattering and did not consider the effect of other scattering mechanisms. In fact, many experimental studies found that the main scattering mechanise for the different types of the thermoelectric materials are different. Even for the same kind of the thermoelectric materials, the carrier scattering mechanism likely changes with temperature, carrier concentration, grain sizes, and so on. For example, based on the theoretical analysis and experimental studies, Wang *et al*. demonstrated that the carriers transport is dominated by acoustic phonon scattering for Cr-free sample and a gradual increasing ionized impurity scattering with increasing Cr content in Cr-Doped Ce_*y*_Co_4_Sb_12_^14^. Pan *et al*. successfully synthesized AgPb_*m*_SnSe_2+*m*_ (m = ∞, 100, 50, 25) samples with a rock salt structure. Between ≈160 and ≈400 K, the dominant scattering process of the carriers from acoustic phonon in PbSe to ionized impurity scattering in AgPb_*m*_SnSe_2+*m*_^15^. Tomes *et al*.^16^ showed that the carrier transport is dominated by alloy scattering at high temperatures and neutral-impurity scattering at low temperatures. In additions, studies have shown that temperature exponents *δ* (also called scattering parameters) of mobility changes from 1.5 to −2.5 in p-type silver-doped PbS with *in situ* Ag_2_S nano-precipitates, indicating the complex scattering processes^17^. Therefore, in addition to acoustic phonon scattering, other scattering processes should also be considered.

It is well known that for a large-scale application of thermoelectric generation, it is important to achieve high ZT in bulk materials^18^. For bulk thermoelectric materials, the dominant elastic scattering mechanism are considered to be acoustic phonon scattering^11, 12^ (with the two modes deformation potential and piezoelectric); neutral impurity scattering^16^ and ionized impurity scattering^14, 19^. As mentioned previously, much attention is paid to explore the best electronic structure when carrier scattering is dominated by acoustic phonons in these thermoelectric materials^11, 12^. However, the systematic research of the ideal electronic structure for thermoelectric materials under different scattering mechanisms is lack. We solves the problem by using the theoretical formulation of carrier mobility in III–V semiconductors (There are far too many thermoelectric materials to cover here, so we will take III-V as an example to discuss)^20^.

## Chasmar-stratton theory

In terms of the reduced Fermi energy, ZT can be expressed as^21^

1 $$ZT=\frac{{[\eta -(\delta +\frac{5}{2})]}^{2}}{{(\beta exp(\eta ))}^{-1}+(\delta +\frac{5}{2})},$$

where *η* is the reduced Fermi energy, *δ* is the scattering parameter which varies depending on the type of scattering, for example, the value of *δ* is $-\frac{1}{2}$ for acoustic-mode lattice scattering, *β* is a materials parameter that was first introduced by Chasmar and Stratton^22^. This material parameter *β* is defined by the relation

2 $$\beta =\frac{\frac{2e{\mathrm{(2}\pi {m}^{\ast }{k}_{B}T)}^{\frac{3}{2}}}{{h}^{3}}{(\frac{{k}_{B}}{e})}^{2}T\mu }{{\kappa }_{ph}},$$

where *k*_*B*_ is Boltzmann’s constant, *h* is the Plank’s constant, and *e* is electron charge. Equation (1) shows that, for a given scattering parameter *δ* and reduced Fermi energy *η*, ZT is a monotonically increasing function of parameter *β*. According to Ioffe^23^, Goldsmid^7, 21^, and Mahan^24^, the relationship between the optimal electronic performance of a thermoelectric semiconductor and material parameter *β* can be reduced to:

3 $$\beta \propto \mu {({m}^{\ast })}^{\frac{3}{2}}.$$

The mobility *μ* can be determined by solving Boltzmann equation in the relaxation time approximation as *μ* = $\frac{e\langle \tau \rangle }{{m}_{I}}$ ^25^, where 〈*τ*〉 is the average relaxation time over the electron energies, *m*_*I*_ (m_*I*_ = 3/(1/m_*x*_ + 1/m_*y*_ + 1/m_*z*_)) is the inertial mass. Equation (3) tells us that mobility *μ* is an important parameter for characterizing the transport of charged carriers. It is well known that the relaxation time varies with scattering mechanism. Thus, the mobility not only depends on the bands structure but also on scattering process. In this paper, we will use the *μ* of III-V-semiconductors as an example to discuss the qualitative relationship between the materials parameter *β* and bands structure under the aforementioned elastic scatterings.

## Material parameter β under various scattering mechanism

(1) Deformation potential scattering. At temperature above absolute zero, the vibrating atoms create pressure (acoustic) waves in a crystal. Displacement of the atoms from their lattice sites are induced by crystal vibrations, which induces a modification of the band gap from point to point. Because the crystal is “deformed” at these points, the potential associated is called the deformation potential. The mobility associated with deformation potential scattering^20, 26^ is

4 $${\mu }_{dp}\propto \frac{1}{{m}_{I}{m}_{b}^{\mathrm{3/2}}}.$$

The materials parameter *β* can be expressed as

5 $${\beta }_{dp}\propto \frac{{N}_{v}}{{m}_{I}}.$$

This can be summarized as follows: if acoustic-mode deformation potential scattering is the predominate mechanism, the mobility is proportional to ${m}_{b}^{-\frac{3}{2}}{m}_{I}^{-1}$, and thus $\mu {({m}^{\ast })}^{\frac{3}{2}}$ is proportional to *N*_*v*_/*m*_*I*_, which has been mentioned in the book edited by Goldsmid in early 1964^21^. (2) Piezoelectric scattering. Piezoelectric effect can occur only in semiconductor due to their polar nature. Electrons can suffer scattering with piezoelectric mode of acoustic lattice vibrations^20, 27^. This effect mainly happens at low temperatures where other scattering mechanisms are weak. Interestingly, the mobility for both piezoelectric scattering and polar optical phonon scattering have the similar expression pattern:

6 $${\mu }_{pe}\propto \frac{1}{{m}_{b}^{\mathrm{1/2}}{m}_{I}}.$$

The materials parameter *β* can be expressed as

7 $${\beta }_{pe}\propto \frac{{N}_{v}{m}_{b}}{{m}_{I}}.$$

(3) Neutral impurity scattering. When an electron passes close to neutral atom, momentum is transferred through a process in which the free electrons exchange with a bound electron on the atom. The mobility associated with neutral impurity scattering is calculated as^28^

8 $${\mu }_{ni}\propto \frac{{m}_{b}^{2}}{{m}_{I}}.$$

The materials parameter *β* can be expressed as

9 $${\beta }_{ni}\propto \frac{{N}_{v}{m}_{b}^{\frac{7}{2}}}{{m}_{I}}.$$

(4) Ionized impurity scattering. In quantum mechanics, ionized impurity scattering means the charge carriers are scattered by ionization in the lattice. The mobility associated with ionized impurity scattering is^27^

10 $${\mu }_{ii}\propto \frac{{m}_{b}^{\mathrm{1/2}}}{{m}_{I}[{\rm{l}}{\rm{n}}\mathrm{(1}+y)-\frac{y}{1+y}]},$$

where ${\rm{y}}=\frac{24\varepsilon {m}_{b}{(kT)}^{2}}{{\hslash }^{2}{e}^{2}n}$. The materials parameter *β* can be expressed as

11 $${\beta }_{ii}\propto \frac{{N}_{v}{m}_{b}^{2}}{{m}_{I}[\mathrm{ln}\,\mathrm{(1}+y)-\frac{y}{1+y}]}.$$

Materials parameter *β* considering the four scattering mechanisms are discussed, which shows that *β* has a close relationship with effective mass m_*i*_ (i = x, y, and z) and the degeneracy of the band maxima near the Fermi level under the same scattering mechanism. It is well known that these two physical quantities have a direct relation with the types of electronic structure for a semiconductor.

## The relation between material parameter *β* and the types of band structure for a semi-conductor

In earlier theoretical work, people always assumed spherical Fermi surfaces and a non-degenerate band-edge state with wave vector **k** = 0^29^. In the simple case of a single, parabolic and isotropic band, the Fermi surface is spherical and the effective mass m_*x*_ = m_*y*_ = m_*z*_ is a constant scalar. The conduction band of most III-V compounds have this feature. In many other semiconductors the extremes of bands are off the center of Brillouin zone^29^. The effective mass along different directions m_||_ = m_*x*_, m_⊥_ = m_*y*_ = m_*z*_ can be different in the same valley. Previous studies have shown that the carrier scattering process can be described by a relaxation time for scattering by neutral impurity or acoustical model^30, 31^, and the different in relaxation time is solely due to the difference in effective mass. However, most of semiconductors have the band structure of the “degenerate’’ type^29^ for which the presence of two or more band edge states of the same energy and wave vector causes the energy surface to have more complicated shapes. Generally speaking, these non-simple model have the anisotropy of the scattering processes. Because the complexity of the anisotropic scattering, this paper deals with only isotropic scattering for the spherical and ellipsoidal Fermi surface.

## The Simple Model

As mentioned above, the simple model assumes a non-degenerate band-edge state with wave vector **K** = 0 and spherical Fermi surfaces. This case, N_*v*_ = 1, m_*x*_ = m_*y*_  = m_*z*_, m_*b*_ = (m_*x*_ m_*y*_ m_*z*_)^1/3^ = m_*x*_ = m_*y*_ = m_*z*_, 1/*m*_*I*_ = 1/*m*_*x*_ = 1/*m*_*y*_ = 1/*m*_*z*_. For the different scattering mechanisms, the materials parameter *β* can be expressed as: (1) deformation potential scattering

12 $${\beta }_{dp}\propto \frac{{N}_{v}}{{m}_{I}}=\frac{1}{{m}_{x}}.$$

From the equation, one could conclude that the light effective mass leads to higher thermoelectric performance. (2) piezoelectric scattering

13 $${\beta }_{pe}\propto \frac{{N}_{v}{m}_{b}}{{m}_{I}}=1.$$

Equation 13 shows that the effective mass has no influence on the thermoelectric properties. (3) Neutral impurity scattering

14 $${\beta }_{ni}\propto \frac{{N}_{v}{m}_{b}^{\frac{7}{2}}}{{m}_{I}}={m}_{x}^{\frac{5}{2}}.$$

Equations 14 shows that larger effective mass is beneficial for thermoelectrics, which is consistent with previous conclusions of enhancement of the thermoelectric efficiency by local increasing the density of states over a narrow energy range^32^. (4) Ionized impurity scattering

15 $${\beta }_{ii}\propto \frac{{N}_{v}{m}_{b}^{2}}{{m}_{I}[{\rm{l}}{\rm{n}}(1+y)-\frac{y}{1+y}]}=\frac{{m}_{x}}{{\rm{l}}{\rm{n}}\mathrm{(1}+y)-\frac{y}{1+y}}.$$

when y is much less than 1,

16 $${\beta }_{ii\mathrm{(1)}}\propto \frac{1}{{m}_{x}}.$$

Thus light effective mass leads to higher thermoelectric performance which is consistent with the conclusion drowned from deformation potential scattering. When y is much larger than 1,

17 $${\beta }_{ii(2)}\propto \frac{{m}_{x}}{\mathrm{ln}\,{m}_{x}}.$$

**Figure 1:**
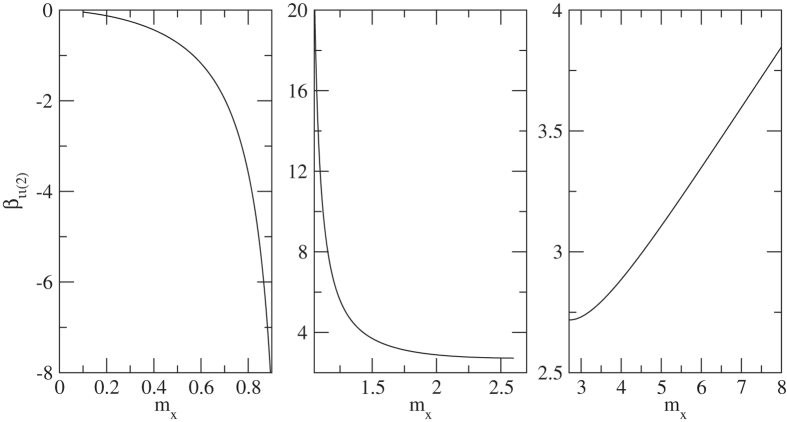
The relative effective mass of *m*_*x*_ dependent materials parameter *β* under ionized impurity scattering when y is much larger than 1. (**a)** 0 < *m*_*x*_ < 1; (**b**) 1 < *m*_*x*_ < 2.7 and (**c**) 2.7 < *m*_*x*_. Note this is a qualitative description of the correlation interactions between *β* and *m*_*x*_.

Figure 1 describes the functional relationship in Equation 17, which indicate that the absolute value of *β* increases with the increasing of *m*_*x*_ when 0 < *m*_*x*_ < 1 and *m*_*x*_ > 2.7, while the value of *β* decreases with the increasing of *m*_*x*_ when 1 < *m*_*x*_ < 2.7. Thus, it is fair to say that the relation between thermoelectric properties and effective mass is different even under the same scattering mechanism.

## The simple many-valley model

As mentioned above, for the simple many-valley model, the carrier scattering process can be described by a relaxation time for scattering by neutral impurity or acoustical model^30, 31^. So ionized impurity scattering are not be discussed here. We can assume that the smallest effective mass is along the x axis, while the effective mass associated with the other two directions are larger. That is: m_*x*_ = *m*_*y*_/*γ* = *m*_*z*_/*γ*, *γ* > 1, where *γ* is anisotropic parameter. In this case, m_*b*_ = ${{\rm{m}}}_{x}{\gamma }^{\frac{2}{3}}$ and 1/*m*_*I*_ = 1/(3*m*_*x*_)(1 + 2/*γ*). No matter which direction of the charge transport along, for the following two scattering mechanisms, the materials parameter *β* can be expressed as: (1) deformation potential scattering

18 $${\beta }_{dp}\propto \frac{{N}_{v}}{{m}_{I}}=\frac{1}{3}\frac{{N}_{v}}{{m}_{x}}(1+\frac{2}{\gamma }).$$

**Figure 2:**
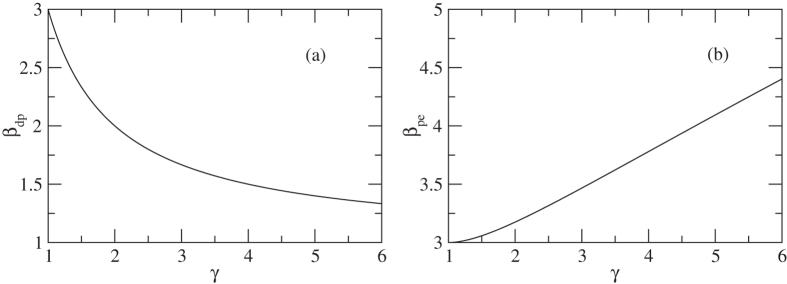
Anisotropic parameter *γ* dependent materials parameter *β*. (**a**) deformation potential scattering; (**b**) piezoelectric scattering. Note this is a qualitative description of the correlation interactions between *β* and *γ*.

This equation tells us that the light band mass and larger band degeneracy contributes to high thermoelectric performance. However, the effect of mass anisotropy on the thermoelectric ZT in the presence of deformation potential scattering is disadvantageous along the principal directions of a small effective mass as shown in Fig. 2(a), which is consistent qualitatively with that of the ionized impurity scattering model^33^. (2) piezoelectric scattering

19 $${\beta }_{pe}\propto \frac{{N}_{v}{m}_{b}}{{m}_{I}}=\frac{{N}_{v}}{3}({\gamma }^{\frac{2}{3}}+\frac{2}{{\gamma }^{\frac{1}{3}}}).$$

(3) neutral impurity scattering

20 $${\beta }_{ni}\propto \frac{{N}_{v}{m}_{b}^{\frac{7}{2}}}{{m}_{I}}\propto {N}_{v}{m}_{x}^{\mathrm{5/2}}({\gamma }^{\mathrm{7/3}}+2{\gamma }^{\mathrm{4/3}}\mathrm{)}.$$

Different from Equations (18), (19) and (20) show that *β* increases with the increasing of anisotropic factor *γ*. Equation (20) also shows that *β* increases with the increasing of the small effective mass m_*x*_. Judging by the Equations (18), (19), and (20), we can draw a conclusion that *β* is a monotonically increasing as a function of the number of degenerate carrier pockets N_*v*_. Generally, this conclusion applies to the systems that already possess multiple bands, achieving the band convergence is beneficial from the m^*^, in which the system still keeps the same group velocity v_*k*_ (a more detailed description can be found in Yang *et al*.)^34^.

From what has been discussed so far, it is obvious that when the carriers are predominantly scattered by the deformation potential scattering, ionized impurity scattering for y ≪ 1, or ionized impurity scattering for y ≫ 1 when $1 < \frac{{m}_{x}}{{m}_{0}} < 2.7$, the light effective mass leads to higher thermoelectric performance, which is applicable to both the simple model and the simple many-valley model. However, when the carriers are predominantly scattered by neutral impurity scattering or ionized impurity scattering for y ≫ 1 when $\,0 < \,\frac{{m}_{x}}{{m}_{0}}\,\mathrm{ < }\,1$ or 2.7 < *m*_*x*_, *β* increases with the increasing of effective mass. Interestingly, when the carriers are predominantly scattered by piezoelectric scattering, the effective mass has nothing to do with thermoelectric properties. It should be noted that, although there are several studies on the relations between the effective mass and thermoelectric properties, none of these studies are concerned with III-V semiconductor^10, 35–38^. For example, Pei *et al*. studied the thermoelectric figure of merit for two groups of La- and I-doped PbTe in the case of potential scattering and found that higher figure of merit in I-doped sample comes from its lower effective mass over the whole temperature considered^10^. This also suggested that the previous conclusion, although derived from III-V semiconductor, is also applicable for other thermoelectric materials. No matter what kind of scattering mechanisms, a larger valley degeneracy is beneficial for thermoelectric performance. This conclusion is in good agreement with many of the previous research on III-V compound semiconductors, as well as other classes of compound semiconductors^11, 39–42^. For example Li *et al*. have shown that convergence of many valleys in the valence band may lead to a high Seebeck coefficient, and induce promising thermoelectric performance of p-type InN^41^. The high band degeneracy and low band effective mass contribute to a high power factor for p-type RuTaSb alloys^35^. It is worth noting that for simple many-valley model, the materials parameter *β* decreases with increasing the anisotropic factor *γ* for deformation potential scattering, however the ZT increases with the increasing of anisotropic factor *γ* for piezoelectric scattering or neutral impurity scattering. Therefore, people used to say that both band effective mass anisotropy and small band effective mass are advantageous to improving the thermoelectric properties, which is derived from specific scattering mechanism^10, 43–50^.

## Conclusion

In summary, we studied the relationship between the thermoelectric properties and the electronic band structure under the assumption of isotropic elastic scattering. The main conclusions can be summarized as follows: (1)About the relation between thermoelectric properties and effective mass: (a) When the carriers are predominantly scattered by the deformation potential scattering, ionized impurity scattering for y ≪ 1 and ionized impurity scattering for y ≫ 1 when $1 < \frac{{m}_{x}}{{m}_{0}} < 2.7$, the light effective mass leads to higher thermoelectric performance, which is applicable to both the simple model and the simple many-valley model; (b) When the carriers are predominantly scattered by neutral impurity scattering and ionized impurity scattering for y ≫ 1 when 0 < *m*_*x*_ < 1 and 2.7 < *m*_*x*_, *β* increases with the increasing of effective mass; (c) When the carriers are predominantly scattered by piezoelectric scattering, the effective mass has nothing to do with thermoelectric properties. (2) About the relation between thermoelectric properties and anisotropy, ZT decrease with increasing the anisotropic factor *γ* for deformation potential scattering, while the materials parameter *β* increase with increasing the anisotropic factor *γ* for piezoelectric scattering and neutral impurity scattering. (3) About the relationship between thermoelectric properties and the degenerate carrier pockets, we found that a larger valley degeneracy is good for thermoelectric materials regardless of scattering mechanism. Further work is required to see if these conclusions are applicable to other thermoelectric performance with more complex Fermi surface under the consideration of the other scattering mechanisms.
